# Analysis of the Causes of Split Pit in Peaches

**DOI:** 10.3390/ijms26125460

**Published:** 2025-06-06

**Authors:** Zhibo Yu, Honghao Huang, Shuangxin Cao, Qi Wang

**Affiliations:** School of Life Sciences, Zhengzhou University, 100 Science Avenue, Zhengzhou 450001, China; hsdyuzhibo@163.com (Z.Y.);

**Keywords:** peach, split pit, influence factors, molecular mechanisms, preventive measures

## Abstract

Split pit is a key factor affecting the quality and yield of peaches; it refers to the failure of the endocarp to close along the suture line. There are significant cultivar-specific differences in peaches regarding their susceptibility or resistance to split pit. During fruit ripening, the stable lignification of the endocarp is crucial to prevent split pit. Excessively rapid and unstable fruit development caused by extreme environments seriously affects endocarp formation, leading to split-pit traits. The uneven distribution of phytohormones is one of the important reasons for uneven fruit growth and split pit. The mature dehiscence model of *Arabidopsis thaliana* provides important references for studying the molecular mechanism of split pit in peaches. This review summarizes the occurrence time, fruit characteristics, influencing factors, and existing research progress on the molecular mechanisms of peach split pit. Finally, it introduces the latest technical methods for studying the molecular mechanisms of split pit and provides future research directions. This paper aims to help breeders understand the possible causes of split pit in peaches, provide a theoretical basis for the prevention and control of split pit, and promote the sustainable development of the peach industry.

## 1. Introduction

Peaches (*Prunus persica* L.), originating from China, are some of the most widely cultivated Rosaceae trees worldwide, only exceeded by apples, grapes, and pears in terms of worldwide production quantity [1,2]. However, compared to other Rosaceae fruits, peaches are prone to pit splitting, which affects the quality and yield of peaches and causes serious economic losses. Split pits are usually caused by a rapid increase in the peach fruit during development; the pressure exerted by the expanding flesh leads to the pit cracking along the suture line [3,4]. Split-pit fruit is more prone to deterioration than healthy fruit, and there is a high risk of disease spread from split-pit fruit to healthy fruit. In recent years, studies on environmental, physiological, genetic, and molecular aspects related to split pits in peaches have been performed. In this review, we summarize the phenomenon of split pit in peaches, the influencing factors, and research directions for the future.

## 2. Phenomenon of Split Pit in Peaches

### 2.1. Fruit Structure of Peaches

A peach fruit develops from an ovary and is classified as a true fruit, which comprises the exocarp, mesocarp, endocarp, and seeds. The exocarp, which is composed of the epidermis and the adjacent layer of flesh cells, is the outermost layer of the fruit. The mesocarp, which is the fleshy portion and the primary edible component of the fruit, manifests as phloem in the early developmental stages of the fruit and gradually transforms into vesicularized thin-walled tissue. The endocarp cells undergo a process of lignification during the splitting and differentiation stages, eventually forming a hard fruit core. For example, in ‘*Okubo*’ peaches, the endocarp begins to lignify about four weeks after flowering, and the lignification process advances from the inside to the outside [5].

### 2.2. Period of Split Pit Occurrence in Peaches

**Figure 1:**
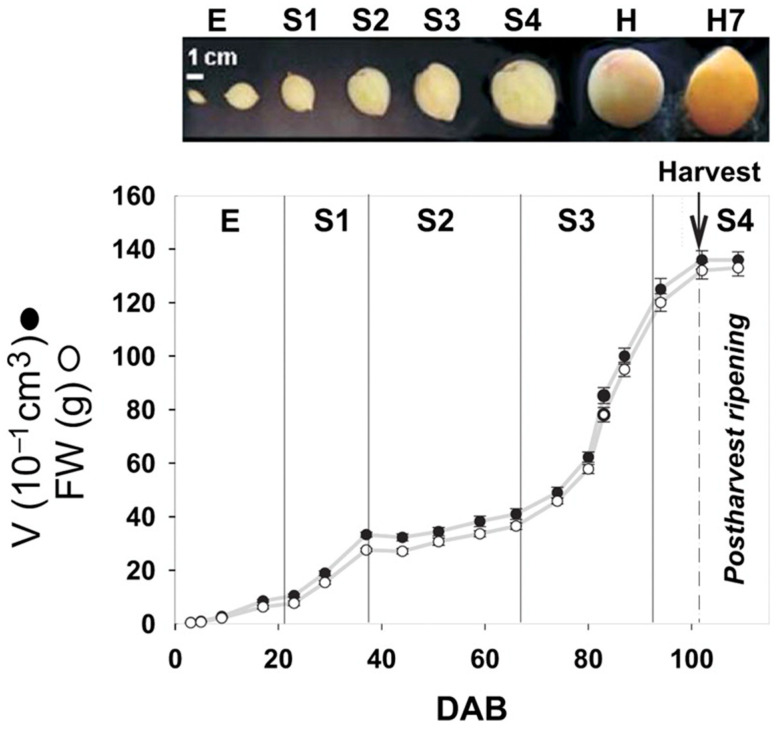
Fruit growth curve of Dixiland peaches [13].

The developmental process of peach fruit can be divided into four stages with a double S-shaped growth curve (Figure 1) [6,7]. Stage 1 (S1) is the rapid growth period, which occurs from approximately 23 to 37 days after blooming (DAB). This stage is characterized by accelerated cell division and elongation, rapid fruit growth, and an increase in fruit size by more than three times. Stage 2 (S2) is the period of fruit lignification, also known as pit hardening, which occurs from about 38 to 66 DAB. In Stage 2, the lignification of the endocarp occurs and the fruit grows more slowly [8]. Stage 3 (S3) is the second exponential growth stage, which occurs from about 67 to 94 DAB. During this stage, cell division accelerates rapidly and the fruit size increases threefold in less than 30 days. In the fourth stage (S4), occurring at approximately 94 to 102 DAB, the fruit reaches its final mature size and enters the ripening period. In the postharvest period, H (Harvest)-H7, the fruit continues to ripen in an ethylene-dependent manner [9]. Split pit usually occurs 2–4 weeks after pit hardening (PH) [10]. During the hardening and lignification process, the pit progressively loses flexibility and becomes extremely rigid, while the mesocarp remains tightly attached. As the fruit expands, the flesh exerts tensile forces on the pit. If these forces exceed the pit’s mechanical strength, cracking occurs along the suture (the structurally weakest region). This is similar to the fruit-splitting model of *Arabidopsis thaliana*, in which the valves break down due to internal tension generated when the fruit dries [11]. Additionally, improper cultivation practices during pit hardening (e.g., premature fruit thinning, excessive irrigation, over-fertilization, gibberellin application) can disrupt the growth coordination between the inner and outer endocarp cell layers, leading to pit splitting [12].

### 2.3. Characteristics of Split-Pit Fruit

Peaches with split pits have a number of distinguishing characteristics. In general, peach fruits with pit splitting exhibit a greater size and mass compared to fruits with unsplit pits. Fruits with cracked pits ripen earlier and soften faster compared to normal fruits [14,15]. Cracked-pit fruits tend to expand irregularly and change in fruit shape. A comparative analysis of the shape index and growth index of sampled nectarines at around 90 DAB revealed that cracked-pit fruits had a higher degree of growth inhomogeneity than normal fruits [16]. In terms of appearance and color, split-pit fruits are usually a morbid yellow-green, and their color is much dimmer compared with that of normal fruits [15,17].

During the gradual ripening of peaches, there is an increase in sugar content. However, cracked-pit fruit is not yet fully developed, resulting in a lower sugar content and, consequently, a relatively bland taste. With respect to the composition ratio, cracked-pit fruit is distinguished by its low sugar content and high acid content, exhibiting a comparatively low sugar–acid ratio. On the contrary, the normal fruit is characterized by its high sugar content and relatively low acidity, resulting in a more pronounced sweetness and a slightly weaker acidity. This profile aligns more closely with the general taste preferences of the public [18].

Previous studies have found that the majority of split-pit fruits grow in a higher position or the top part of the tree, where they have advantages in nutrient and water uptake, thus accelerating the growth and development process of the fruit. However, the phenomenon of split-pit is very easily caused. As the fruit can fully access nutrients, sometimes a nutrient excess will lead to pit splitting [14]. In addition, most split-pit fruit is cracked at the pedicel and may also develop mold spots. More seriously, the fruit will begin to dry up gradually from the split, eventually resulting in the complete drying out of the entire fruit [17]. At the same time, split-pit fruits are usually accompanied by the phenomenon of fruit cracking. This occurrence can be attributed to the generation of strong tension within the pit due to its splitting. This tension leads to the outward opening of the flesh, resulting in the observed splitting of the fruit [8].

The pits of split-pit peaches grow significantly faster than normal cultivars throughout development, and this rapid growth may result in a less dense pit structure, increasing the risk of splitting. Meanwhile, the seeds of split-pit cultivars have faster extension than the seeds of normal cultivars, but after the middle stage of hardening, the dry matter accumulation is slower, resulting in poorer coordination between seed development and pit hardening. Poor seed development will exacerbate pit splitting [19].

## 3. Factors Affecting Split Pit in Peaches

### 3.1. Germplasm Characterization

**Figure 2:**
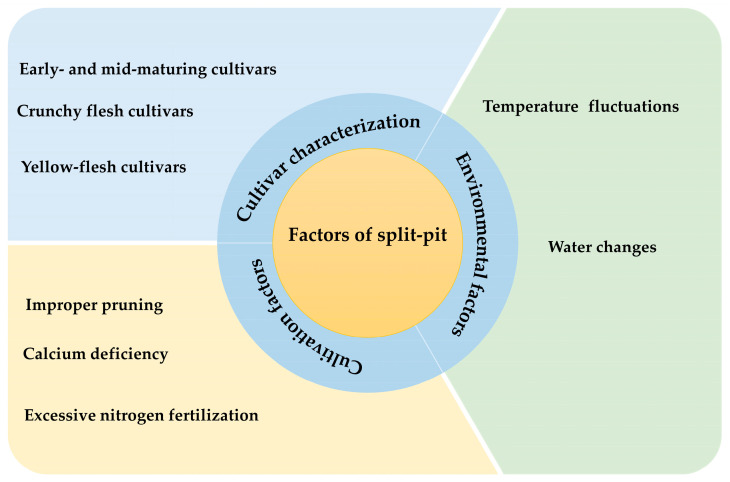
Factors affecting split-pit peaches.

There are significant differences in the susceptibility of different peach cultivars to pit splitting (Figure 2). Some cultivars are susceptible to pit cracking because of the fragile structure of the pit due to their genetic characteristics. For example, crunchy flesh cultivars are more prone to pit splitting than dense flesh cultivars, partially round cultivars are more prone to pit splitting than oblong ones, and early- and medium-ripening cultivars are more prone to pit splitting than late-ripening cultivars. Moreover, the peach pit contains seeds, usually two seeds inside each fruit, which produce more expansion force than a single seed, and therefore, these fruits are more likely to produce pit splitting.

Nectarines have a higher incidence of split pit than melting-flesh peaches, and yellow-flesh cultivars have a higher incidence of split pit than white-flesh cultivars. Drogoudi et al. [20] revealed that the split-pit incidence of the cultivars ‘Alitop’ and ‘Francoise’ was the highest, reaching 61.6%. Cultivars with lower split-pit rates belonged to white-flesh melting peaches, such as ‘Gladys’, ‘Oetavia’, and ‘Rosalia’. Selecting these genotypes for cultivation could effectively mitigate substantial economic losses in commercial orchards. This may relate to the more volatile expression of split-pit-associated genes and the lower lignin deposition efficiency in yellow-flesh cultivars. However, white-flesh cultivars show higher split-pit rates under high nitrogen fertilization, suggesting that nitrogen disrupts ethylene synthase gene (ACO) expression to override inherent resistance. This highlights genotype–environment interactions masking simple cultivar traits, limiting screening based solely on flesh color or maturity [17]. Additionally, split-pit resistance often correlates negatively with fruit size and the sugar–acid ratio. This negative correlation compels breeders to balance split-pit resistance against high-quality and large-fruit traits during cultivar development.

### 3.2. Environmental Factors

Water is a key environmental factor influencing pit splitting in peaches. During the pre-expansion stage of the fruit, when soil moisture is unstable, especially after an initial drought, and the fruits are then suddenly exposed to rainfall or overwatering during the expansion stage, the fruit will rapidly absorb water and expand. The internal pressure increases, which will lead to pit splitting. Rapid fluctuations in temperature can also induce the uneven expansion or contraction of endocarp tissues, thereby triggering pit splitting [15,20].

### 3.3. Cultivation and Management Factors

Improper fertilization is one of the most important cultivation and management factors that contributes to pit splitting in peaches. Excessive nitrogen fertilization accelerates the development of ventral vascular bundles, deepening and widening the endocarp grooves while reducing their mechanical robustness, thereby increasing the susceptibility to splitting [17]. Calcium is an important element for maintaining cell wall stability, and a calcium deficiency will lead to a decrease in cell wall strength, thus increasing the risk of split pit. A low calcium concentration in the pit and flesh may be one of the important factors leading to pit splitting [21]. Field studies have demonstrated that foliar applications of calcium–sugar alcohol chelate solutions during fruit development significantly reduce pit cracking, enhance fruit quality, and elevate the soluble sugar content [22]. Additionally, inadequate pruning practices that expose fruits to direct solar radiation may induce thermal stress, exacerbating the split-pit susceptibility through the temperature-mediated structural weakening of the endocarp.

## 4. Molecular Mechanisms of Split Pit in Peaches

### 4.1. Endocarp Development and Lignification: Structural and Physiological Foundations in Peaches

Following fertilization, floral structures undergo significant morphological changes, with the ovary and its associated tissues developing into a fruit. The pericarp, derived from the ovary wall, encases the seeds. Some endocarps have many large and numerous juice sacs, such as those of citrus fruits and grapefruits; some have cells that separate into a sauce as the fruit ripens, such as that of grapes; and some are rigid and consist of stone cells that form a hard core, such as those of peaches, plums, and coconuts. Peaches are fleshy, single fruits that develop from a single carpel, whose endocarp develops mainly from the inner wall of the ovary [23]. The endocarp lignifies to form a protective structure around the seed, and the lignified endocarp protects the seed from disease or digestion by herbivores [24,25]. The lignification of the endocarp represents an effective strategy for drupes to protect seeds. However, if the lignification process is profoundly influenced by external environmental fluctuations, such as drastic changes in moisture and temperature, endocarp lignification becomes unstable. This instability consequently disrupts the overall fruit development and induces pit splitting.

In peach fruit, the peach pit has a high lignin content, with a seasonal pattern of lignin accumulation [26]. Moreover, lignin accumulation in a peach pit exhibits a distinct inside-to-outside gradient during this process [27].

During the early development stage of the peach fruit endocarp, abundant phenolic and tannin-like substances are present among thin-walled cells, which may represent the early deposition of lignin. Additionally, peach fruits contain high levels of polyphenols and amino acids (e.g., alanine, proline, glutamine, and phenylalanine), which are utilized in phenylalanine and lignin biosynthesis pathways during pit hardening [13]. In the later stage of pit hardening, thin-walled cells begin to differentiate into sclereid cells. Their cell walls thicken significantly, organelles degrade, cellular inclusions reduce, large intercellular spaces form, and numerous vesicles and reticular structures appear, possibly as byproducts of organelle breakdown. Eventually, the endocarp matures into a rigid shell through this lignification cascade [28]. Lignin formation relies on multiple primary metabolites, with phenylalanine being the most critical. If a primary metabolite is deficient or excessive in the fruit, it may disrupt the formation of stone cells, ultimately contributing to the occurrence of split-pit fruits. Investigating the relationship between primary metabolites and split-pit fruits using novel techniques such as metabolomics is, therefore, of great significance.

During the developmental process of peach fruits, endocarp lignification is one of the key features. By investigating lignin-related metabolic pathways and the roles of enzymes, we can gain a better understanding of the endocarp development process and the mechanisms underlying split-pit formation. Biochemical analyses of drupes such as olives, black walnuts, peaches, and coconuts have revealed that their endocarps contain more than twice the lignin content of wood, indicating a complex mechanism underlying secondary wall biosynthesis in this tissue [29]. During the pit-hardening phase (Stage 2) of the double S fruit growth curve, competition between embryo growth and endocarp lignification induces the temporary cessation of mesocarp expansion, which coincides with the onset of endocarp lignification [30]. Notably, in early-maturing peach and plum cultivars, the second exponential growth phase (S3) begins before the endocarp is fully lignified [31,32].

Ryugo first identified lignin in peach pits in 1961. Lignin, a plant-specific polyphenolic polymer, plays a critical role in tree crops for applications in paper production, animal feed, and biofuel industries, underscoring its economic significance [33]. Subsequent studies have identified peroxidases and phenolic oxidases as key enzymes in lignin biosynthesis [34], with lignin formation involving the sequential action of phenol oxidases, peroxidases, and laccases [35]. In 2010, Dardick et al. [36] observed the lignification process of peach endocarps through the use of gene chip technology, and genes related to the phenylalanine metabolism pathway, lignin formation, and flavonoid synthesis are induced at the same time.

In peach endocarp cells, phenol oxidase is predominantly associated with ion-bound cell wall proteins, suggesting its involvement in lignin monomer polymerization. This observation implies the enzyme’s role in early lignified cell wall modifications, such as oligomeric lignin polymerization at the end of the rapid growth phase [37]. Peroxidase and laccase, conversely, are more closely linked to the later stages of hardening, where their activities correlate with increasing lignin content during the second fruit development stage [36]. The aforementioned findings provide critical insights into the physiological mechanisms of endocarp lignification in peaches, laying the groundwork for molecular studies on split-pit formation. Key metabolic enzymes play a pivotal role in endocarp lignification, and their expression and activity are closely associated with the split-pit mechanism. Deepening our understanding of endocarp formation is, therefore, critical for unraveling the molecular basis of peach split pit.

### 4.2. Effect of Phytohormones on Split Pit in Peaches

Phytohormones play key regulatory roles in peach fruit development, with indole-3-acetic acid (IAA) acting as a primary growth promoter during both the rapid growth and cell expansion phases. IAA promotes fruit enlargement by inducing the expansion of mesocarp parenchyma cells. Ethylene, another key phytohormone, regulates fruit maturation and induces the over-ripening and subsequent softening of fruits and vegetables [38]. While IAA is abnormally expressed during the pit-hardening stage, it is likely to affect the lignification process of parenchyma cells. The dynamic balance of IAA and ethylene is closely related to pit cracking, and the uneven distribution of IAA and ethylene in split-pit fruits may ultimately lead to endocarp dehiscence by promoting the uneven growth of the fruit flesh. It has been shown that a low IAA content in the dehiscence zone is necessary for dehiscence occurrence [39]. Normal fruits, in contrast to pit-split fruits, show a less marked imbalance in IAA and ethylene distribution, especially at the central suture, where the hormone concentrations are lower. Conversely, the ethylene levels in split-pit fruits are, on average, 11.6 times greater than those in normal fruits [16]. The content of IAA and ethylene in different fruit regions is pivotal for phenotypic changes, and a mistimed overexpression of these phytohormones represents a critical factor in split-pit development.

As a key driver of cell expansion, indole-3-acetic acid (IAA), when disproportionately accumulated in the mesocarp, accelerates cellular elongation, causing the lateral growth rate of the mesocarp to outpace the hardening rate of the endocarp and inducing mechanical tension on the pit. Elevated IAA concentrations up-regulate ethylene biosynthesis precursors, triggering a “hormonal positive feedback loop” that amplifies the risk of mesocarp overgrowth and endocarp fracturing [40]. Wu et al. [41] found that hormone signal transduction was the most enriched category of differentially expressed genes, indicating that the initiation and progression of endocarp lignification in peaches require substantial hormonal mobilization. Therefore, minimizing the hormonal impacts on split-pit formation holds significant practical value for controlling the split-pit incidence.

### 4.3. Progress on Split-Pit Gene

**Figure 3:**
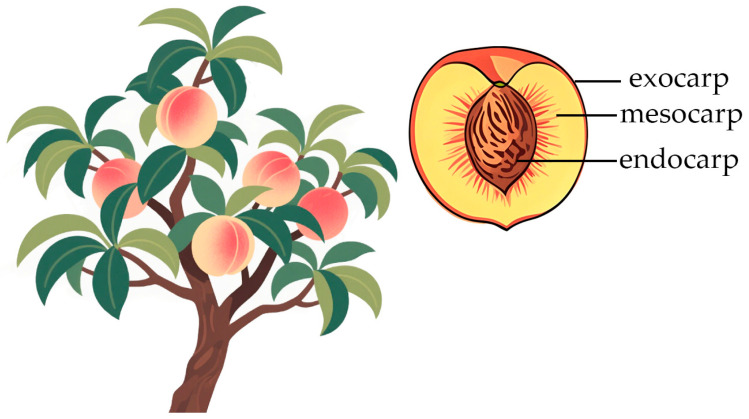
Structure of peach fruit.

There are many similarities between peaches and *Arabidopsis thaliana* in terms of the anatomical structure and physiological function of their flowers and fruits (Figure 3). Specifically, the peach pericarp resembles the valve organ (valve margin) of *Arabidopsis*, both of which originate from the carpel tissue of the ovary, and cleavage in both occurs in the region of the separating layer of the endocarp [11].

The expression and interactions of the *SHP* and *FUL* genes in *Arabidopsis* has been a focus of extensive investigation [42,43]. *SHP1/2* belong to the *AGAMOUS (AG)* clade [44]. The molecular mechanism of *Arabidopsis* fruit dehiscence was first described by Liljegren et al. [45] in 2000. *SHP1/2* are expressed during early carpel development and regulate cell differentiation in the dehiscence zone (DZ). Mutants in which both genes are knocked out fail to develop a functional DZ, exhibiting reduced lignification and an ill-defined separation layer. This situation prevents proper fruit dehiscence under desiccation [46]. Conversely, *Arabidopsis* overexpressing *SHP1* or *SHP2 (35S::SHP1* and *35S::SHP2)* show enhanced valve lignification, accelerating fruit dehiscence [47]. The relationship between the *SHP/FUL* genes and key enzymes in the lignin metabolic pathway (phenol oxidases, peroxidases, and laccases) likely represents a critical component of the split-pit mechanism.

*FUL* belongs to *APETALA1 (AP1)/FUL*. Ferrándiz et al. [48] demonstrated that *FUL* antagonistically regulates *SHP* expression. In loss-of-function *FUL* mutants, *SHP* is ectopically expressed in the valve tissues, leading to premature lignification, whereas in *FUL*-overexpressing lines, the valve margin cells are reprogrammed into a valve mesophyll cell fate, with adjacent separation zone cells failing to lignify, ultimately impairing fruit dehiscence [49]. The *SHP/FUL* genes are members of the MADS-box transcription factor family, and they likely exert a significant influence on the lignin metabolic pathway.

In the genetic mechanism that regulates the formation of the DZ, *SHP* is placed at the top of the genetic hierarchy that directs the formation of the DZ, whereas *FUL* acts on the ovary wall to restrict *SHP* expression domains and, thus, ensure the correct spatial localization of the DZ [50]. The *SHP* genes are expressed at the valve margins from the early stages of gynoecium development, where they activate *IND* expression. *SHP* is necessary for both the septum and lignum development, and *ALC* is required for separation layer formation. *FUL* is expressed at the valve, where it represses *SHP* and *IND* expression, while *RPL* plays the same role in the septum. Thus, *FUL* and *RPL* restrict *SHP*, *ALC*, and *IND* expression to narrow strips of cells that will differentiate into DZ at the edge of the valve [51].

### 4.4. Split-Pit Genes of Peaches

The peach pit-splitting process is similar to that of *Arabidopsis* angiosperm dehiscence, and the researchers hypothesized that the *PPERFUL* (*FRUITFULL* homologue) and *PPERSHP* (*SHATTERPROOF* homologue) genes in peach fruits may play important roles in the formation of split pit. The peach pit-splitting phenomenon mainly occurs during the pit-hardening and cell enlargement stages of fruit development, and its molecular mechanism is closely related to the regulation of the spatial and temporal expression of the *PPERFUL* and *PPERSHP* genes. Tani et al. [49] showed that *PPERFUL* was expressed to some extent during the early stage of fruit development (1–4 weeks after full blooming), decreased significantly during pit hardening (7–11 weeks after full bloom), and increased again during the final stage of fruit growth (12–15 weeks after full bloom). Notably, the decline in *PPERFUL* expression at pit hardening was greater in the split-pit-susceptible cultivar (‘Andross’), whereas the expression was relatively stable at this stage in the split-pit-resistant cultivar (‘Katherine’). This expression pattern implies that *PPERFUL* may promote split-pit formation by influencing the process of pit lignification. Due to insufficient lignin deposition and the insufficient structural strength of the pit, this may lead to an exacerbation of the growth imbalance between the flesh and the pit in split-pit-susceptible cultivars [49]. In the *Arabidopsis* model, *FUL*-deficient mutants exhibited ab enhanced formation of lignified cells, even leading to valve lignification, instead of the insufficient lignin deposition observed in split-pit-susceptible cultivars.

Tani et al. [49] showed that the expression pattern of another key gene, *PPERSHP*, was closely related to pit splitting. During pit hardening, *PPERSHP* expression was significantly lower in split-pit-resistant cultivars than in split-pit-susceptible cultivars. Combined with the *Arabidopsis* research model, *PPERSHP* may be involved in the formation of the endocarp separation layer by activating the lignification process of cells in the dehiscence zones. The low expression of *PPERSHP* in split-pit-resistant cultivars may have inhibited excessive lignin deposition, enhanced pit flexibility, and reduced the risk of dehiscence along the ventral suture line, further supporting a direct correlation between differences in gene expression and the pit structural stability. In addition, lignin staining showed that endocarp lignification was higher in split-pit-resistant cultivars at the early stage of pit hardening. The stable formation of the endocarp during the early stage of hardening is more critical for the subsequent stable development of the fruit pit. The dynamic and balanced expression of *PPERFUL* and *PPERSHP* regulates the synchronization of pit hardening and flesh growth. An imbalance in the expression of these two genes may lead to an insufficient mechanical strength of the endocarp or the premature formation of dehiscence regions, which ultimately triggers pit splitting [49].

*AGAMOUS*, *SEEDSTICK*, *SEPALLATA1*, *SEPALLATA3*, and *FBP9* are key regulators of floral and fruit development [52]; they belong to the CDE class of the ABCDE floral organ identity model. Their peach homologs, including *PERAG*, *PPERSTK*, *PPERSEP1*, *PPERSEP3*, and *PPERFBP9*, exhibit distinct roles in endocarp development. *PPERAG* belongs to the class C genes, which mainly regulate carpel and ovule development and endocarp lignification, and it is stably expressed in split-pit-resistant cultivars, while its expression in split-pit-susceptible cultivars fluctuates greatly during pit hardening. The down-regulation of this gene in split-pit-susceptible cultivars may lead to insufficient lignin deposition and reduce the mechanical strength of the pit. *PPERSTK* belongs to the class D genes, which mainly regulate ovule development and endocarp lignin deposition, and this gene is specifically expressed in the carpels and ovules. This gene is expressed earlier and more persistently in split-pit-resistant cultivars, while the peach fruits of split-pit-susceptible cultivars show a significant decrease in expression at the pit-hardening stage. The low expression of this gene leads to a delay in lignification and exacerbates the fragility of the endocarp structure. *PPERSEP1*, *PPERSEP3*, and *PPERFBP9* belong to the class E genes, which are mainly involved in the regulation of flower organ development and fruit ripening. All of these genes are stably expressed in split-pit-resistant cultivars, while their expression is absent in split-pit-susceptible cultivars during pit hardening [11]. These genes can help avoid the mechanical stress caused by over-expanding flesh by maintaining coordination between flowering and fruit development. If expressed abnormally in split-pit-susceptible cultivars, these genes may lead to an imbalance between flesh and pit growth, which, in turn, triggers split pit. These mechanisms provide a direct theoretical basis at the molecular level for the selection of split-pit-resistant cultivars and the optimization of cultivation measures.

**Figure 4:**
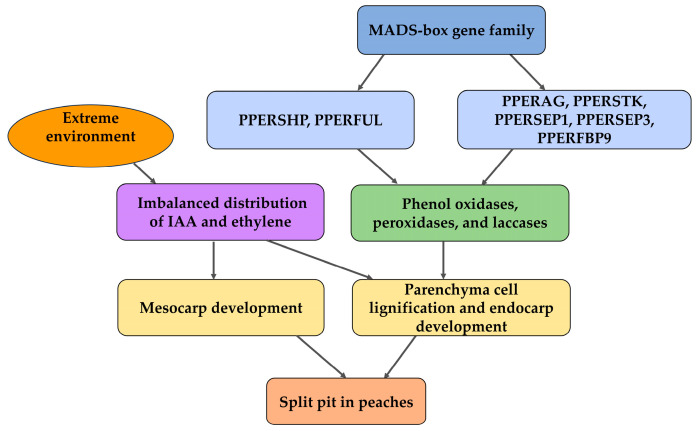
The effects of split-pit-related genes and phytohormones on the split-pit phenotype.

The MADS-box gene family is a crucial regulator of floral and fruit development. Among them, *PPERFUL* and *PPERSHP* have the most direct impact on endocarp lignification. These genes, together with *PPERAG*, *PPERSTK*, *PPERSEP1*, *PPERSEP3*, and *PPERFBP9*, may influence the expression and activity of lignification-related enzymes, thereby affecting endocarp development. Extreme external environments can alter the expression levels and distribution of phytohormones in fruits. Phytohormones not only regulate the endocarp lignification process, but also affect mesocarp development. The excessive growth of the mesocarp generates tensile forces on the endocarp, leading to severe split pit (Figure 4). Understanding how MADS-box-family genes and phytohormones impact endocarp lignification may hold the key to unraveling the mechanisms of split-pit formation.

## 5. Prospects of Molecular Breeding Technologies for Split-Pit Resistance

### 5.1. Transcriptomics and Candidate Gene Mining

Transcriptomics includes the functional genome of living organisms, incorporating the total number of transcripts, their abundance in a specific cell, and post-transcriptional modifications [53]. Transcriptome profiling is dynamic and has emerged as a promising technique for analyzing gene expression in response to stimuli over a specific time period. By capturing differentially expressed genes (DEGs) across developmental stages or under stress, this technique has become a core tool for dissecting the genetic basis of fruit quality traits [54]. For example, Liu et al. [55] selected two exocarp periods (19 and 69 DAB) for transcriptome sequencing. Transcriptome analyses were used to identify the genes associated with fruit pigmentation and chlorophyll (Chl) degradation in peach fruit skins. The transcriptome analysis identified 3414 up-regulated genes and 3126 down-regulated genes. An analysis showed that the *PpCLH2* gene and *NYC1*-like may also play important roles in fruit chlorophyll degradation. In the study of single-fruit weights in peaches, differentially expressed genes were identified through an RNA-seq data analysis. By combining dynamic phenotypic changes, the researchers screened out three candidate genes related to auxin signaling. This discovery offers a novel perspective for revealing the genetic mechanism underlying single-fruit weights in peaches [56]. By comparing the transcriptome data of the endocarp at different developmental stages between split-pit-resistant and -susceptible peach cultivars, differentially expressed genes associated with split pit would be identified.

### 5.2. QTL Mapping and Multi-Trait Breeding

Modern breeding technologies and the development of quantitative trait locus (QTL) mapping have brought about a new era in peach breeding [57]. This genetic method pinpoints specific areas of the genome that are closely associated with characteristics such as the fruit shape, color, size, or flavor [58]. As a quality trait that seriously impacts fruit commerciality, split pit can be studied through QTL mapping technology. This technology not only identifies genetic intervals significantly associated with the split-pit phenotype, but also uncovers key genes regulating split-pit occurrence. These findings enable the development of molecular markers, thereby accelerating the breeding process. QTL mapping is capable of discovering genes associated with numerous phenotypes, which is an important phenomenon when breeding plants with a variety of desired characteristics [59,60]. Another core advantage of QTL mapping lies in the simultaneous improvement of multiple traits. Hernández Mora et al. [61] revealed the genetic basis of seven key agronomic traits by integrating the phenotypic and genotypic data of 18 peach hybrid populations. In addition, they identified a QTL on chromosome 4 (qMD4) that affects both the fruit weight (FW) and the maturity date (MD), demonstrating that the pleiotropy of QTLs can be used to design a convergent breeding scheme for “high-yield and early-maturity” peaches.

### 5.3. CRISPR Editing and Split-Pit Mechanism Research

The technology of gene editing is a powerful tool for functional genomics in improving the quality and commercial value of fruits. The emergence of CRISPR/Cas9 technology provides a new opportunity to accelerate plant molecular breeding. The CRISPR/Cas9 technology used on fruit crops not only provides a shortcut for obtaining a high yield and good quality of fruit food, but also lays a solid foundation for fruit functional genomics research [62]. In tomatoes, Li et al. [63] knocked out *SGR1*, *LCY-B1*, and *LCY-B2* using the CRISPR/Cas9 method, which inhibited the conversion of lycopene and increased the lycopene content in the fruit by about 5.1 times. Pectate lyases (PLs) are important components of pectinase. Uluisik et al. [64] utilized CRISPR/Cas9 to induce mutations in the tomato *PL* gene, thereby enhancing fruit firmness and extending the shelf life without adverse effects on other aspects of fruit ripening [65]. These technological advancements offer critical insights for improving complex traits like split pit. By targeting genes governing pit lignification, CRISPR enables the precise regulation of cell wall metabolism to genetically decode split-pit mechanisms and breed split-pit-resistant varieties, thereby enhancing the peach commercial value.

## 6. Summary

Peach pit splitting is a complex problem involving multiple factors. Although previous studies have been focused on the causes, physiological and molecular mechanisms, and control measures of peach pit splitting, there are still many problems to be further studied. For example, the main effector gene of peach pit splitting has not yet been clarified, and the mechanism of the difference in split-pit susceptibility among different cultivars is not yet clear. Additionally, existing control measures have not addressed the breeding bottleneck of the negative correlation between split-pit resistance and premium quality traits. Future research should prioritize investigating the molecular mechanisms of peach split pit by using genome-wide association studies (GWASs) and spatiotemporal transcriptomics to decipher the co-expression networks of split-pit-related genes in suture abscission layer cells and map major-effect QTLs. CRISPR-Cas9 technology should be employed to edit key enzyme genes involved in lignin synthesis, thereby exploring the impact of lignin biosynthesis on endocarp development and split-pit traits. Establishing efficient genetic transformation systems will facilitate the breeding of dual-purpose cultivars with both split-pit resistance and a superior quality. Meanwhile, optimizing the current control measures and developing more efficient and friendly technologies for split-pit prevention will provide robust support for the sustainable development of the peach industry.

## Data Availability

No new data were created or analyzed in this study. Data sharing is not applicable to this article.
